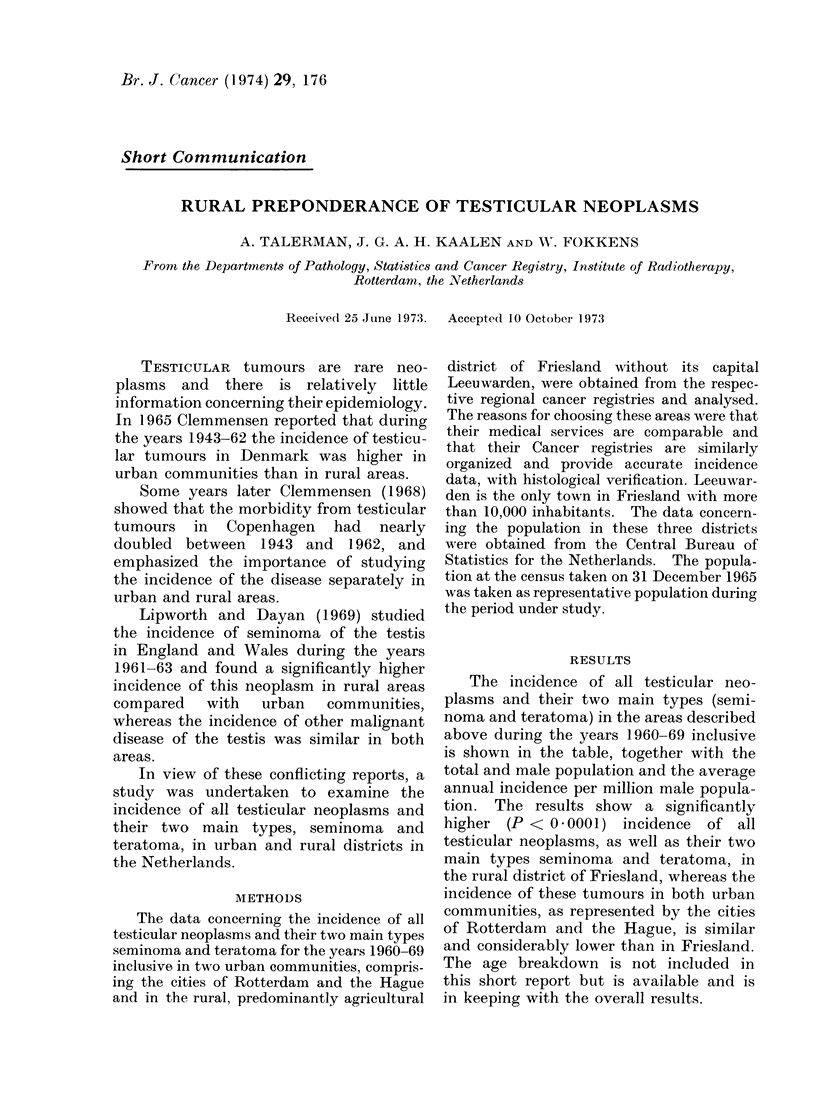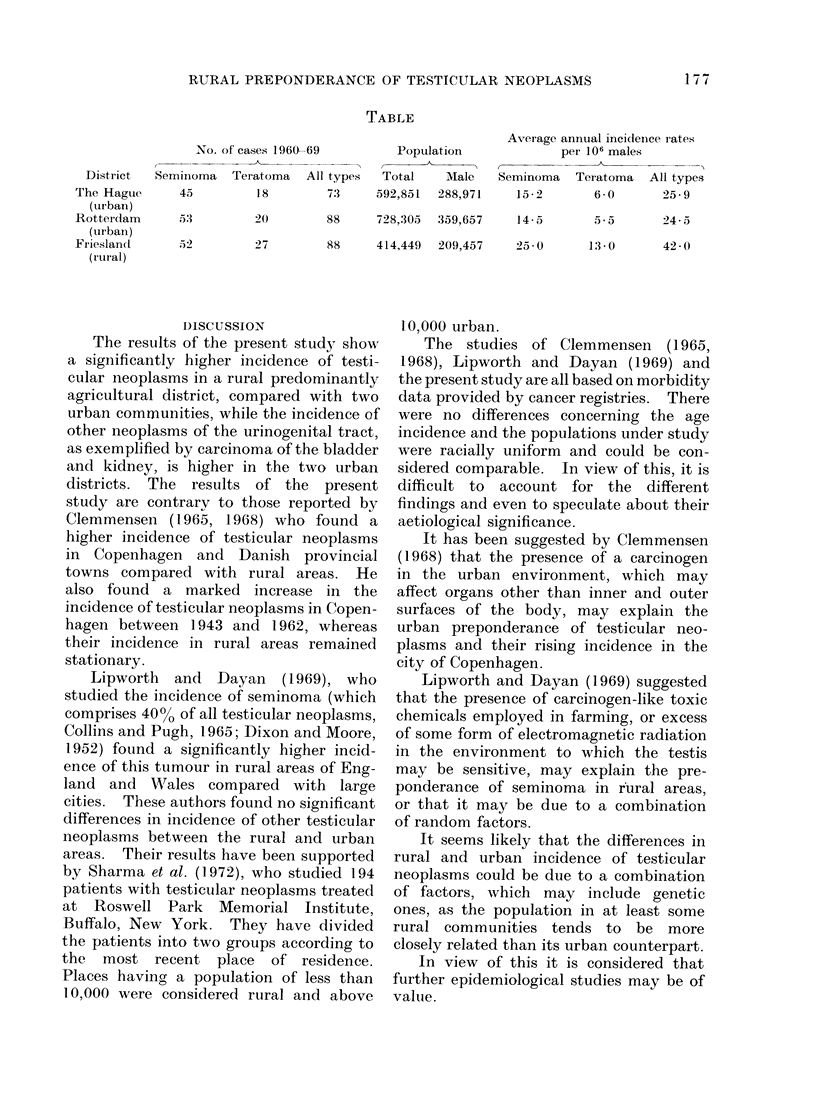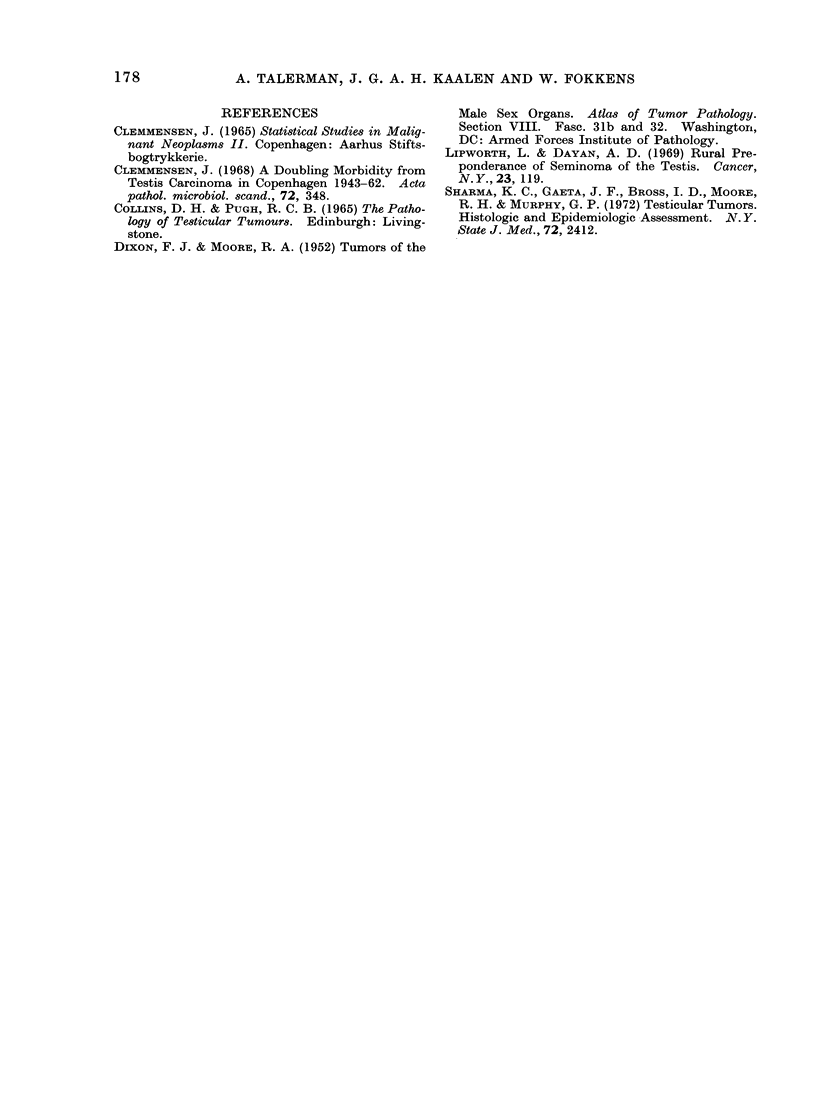# Rural preponderance of testicular neoplasms.

**DOI:** 10.1038/bjc.1974.54

**Published:** 1974-02

**Authors:** A. Talerman, J. G. Kaalen, W. Fokkens


Br. J. Cancer (1974) 29, 176

### *Short Communication*

### RURAL PREPONDERANCE OF TESTICULAR NEOPLASMS

A. TALERMAN, J. G. A. H. KAALEN AND W. FOKKENS

*From the Departments of Pathology, Statistics and Cancer Registry, Institute of Radiotherapy, Rotterdam, the Netherlands*

Received 25 June 1973. Accepted 10 October 1973

TESTICULAR tumours are rare neoplasms and there is relatively little information concerning their epidemiology. In 1965 Clemmensen reported that during the years 1943–62 the incidence of testicular tumours in Denmark was higher in urban communities than in rural areas.

Some years later Clemmensen (1968) showed that the morbidity from testicular tumours in Copenhagen had nearly doubled between 1943 and 1962, and emphasized the importance of studying the incidence of the disease separately in urban and rural areas.

Lipworth and Dayan (1969) studied the incidence of seminoma of the testis in England and Wales during the years 1961–63 and found a significantly higher incidence of this neoplasm in rural areas compared with urban communities, whereas the incidence of other malignant disease of the testis was similar in both areas.

In view of these conflicting reports, a study was undertaken to examine the incidence of all testicular neoplasms and their two main types, seminoma and teratoma, in urban and rural districts in the Netherlands.

### METHODS

The data concerning the incidence of all testicular neoplasms and their two main types seminoma and teratoma for the years 1960–69 inclusive in two urban communities, comprising the cities of Rotterdam and the Hague and in the rural, predominantly agricultural district of Friesland without its capital Leeuwarden, were obtained from the respective regional cancer registries and analysed. The reasons for choosing these areas were that their medical services are comparable and that their Cancer registries are similarly organized and provide accurate incidence data, with histological verification. Leeuwarden is the only town in Friesland with more than 10,000 inhabitants. The data concerning the population in these three districts were obtained from the Central Bureau of Statistics for the Netherlands. The population at the census taken on 31 December 1965 was taken as representative population during the period under study.

### RESULTS

The incidence of all testicular neoplasms and their two main types (seminoma and teratoma) in the areas described above during the years 1960–69 inclusive is shown in the table, together with the total and male population and the average annual incidence per million male population. The results show a significantly higher ($P < 0 \cdot 0001$) incidence of all testicular neoplasms, as well as their two main types seminoma and teratoma, in the rural district of Friesland, whereas the incidence of these tumours in both urban communities, as represented by the cities of Rotterdam and the Hague, is similar and considerably lower than in Friesland. The age breakdown is not included in this short report but is available and is in keeping with the overall results.

TABLE

| District | No. of cases 1960–69 | | | Population | | Average annual incidence rates per $10^6$ males | | |
|---|---|---|---|---|---|---|---|---|
| | Seminoma | Teratoma | All types | Total | Male | Seminoma | Teratoma | All types |
| The Hague (urban) | 45 | 18 | 73 | 592,851 | 288,971 | 15·2 | 6·0 | 25·9 |
| Rotterdam (urban) | 53 | 20 | 88 | 728,305 | 359,657 | 14·5 | 5·5 | 24·5 |
| Friesland (rural) | 52 | 27 | 88 | 414,449 | 209,457 | 25·0 | 13·0 | 42·0 |

### DISCUSSION

The results of the present study show a significantly higher incidence of testicular neoplasms in a rural predominantly agricultural district, compared with two urban communities, while the incidence of other neoplasms of the urinogenital tract, as exemplified by carcinoma of the bladder and kidney, is higher in the two urban districts. The results of the present study are contrary to those reported by Clemmensen (1965, 1968) who found a higher incidence of testicular neoplasms in Copenhagen and Danish provincial towns compared with rural areas. He also found a marked increase in the incidence of testicular neoplasms in Copenhagen between 1943 and 1962, whereas their incidence in rural areas remained stationary.

Lipworth and Dayan (1969), who studied the incidence of seminoma (which comprises 40% of all testicular neoplasms, Collins and Pugh, 1965; Dixon and Moore, 1952) found a significantly higher incidence of this tumour in rural areas of England and Wales compared with large cities. These authors found no significant differences in incidence of other testicular neoplasms between the rural and urban areas. Their results have been supported by Sharma *et al.* (1972), who studied 194 patients with testicular neoplasms treated at Roswell Park Memorial Institute, Buffalo, New York. They have divided the patients into two groups according to the most recent place of residence. Places having a population of less than 10,000 were considered rural and above 10,000 urban.

The studies of Clemmensen (1965, 1968), Lipworth and Dayan (1969) and the present study are all based on morbidity data provided by cancer registries. There were no differences concerning the age incidence and the populations under study were racially uniform and could be considered comparable. In view of this, it is difficult to account for the different findings and even to speculate about their aetiological significance.

It has been suggested by Clemmensen (1968) that the presence of a carcinogen in the urban environment, which may affect organs other than inner and outer surfaces of the body, may explain the urban preponderance of testicular neoplasms and their rising incidence in the city of Copenhagen.

Lipworth and Dayan (1969) suggested that the presence of carcinogen-like toxic chemicals employed in farming, or excess of some form of electromagnetic radiation in the environment to which the testis may be sensitive, may explain the preponderance of seminoma in rural areas, or that it may be due to a combination of random factors.

It seems likely that the differences in rural and urban incidence of testicular neoplasms could be due to a combination of factors, which may include genetic ones, as the population in at least some rural communities tends to be more closely related than its urban counterpart.

In view of this it is considered that further epidemiological studies may be of value.

### REFERENCES

CLEMMENSEN, J. (1965) *Statistical Studies in Malignant Neoplasms II*. Copenhagen: Aarhus Stiftsbogtrykkerie.

CLEMMENSEN, J. (1968) A Doubling Morbidity from Testis Carcinoma in Copenhagen 1943–62. *Acta pathol. microbiol. scand.*, **72**, 348.

COLLINS, D. H. & PUGH, R. C. B. (1965) *The Pathology of Testicular Tumours*. Edinburgh: Livingstone.

DIXON, F. J. & MOORE, R. A. (1952) Tumors of the Male Sex Organs. *Atlas of Tumor Pathology*. Section VIII. Fasc. 31b and 32. Washington, DC: Armed Forces Institute of Pathology.

LIPWORTH, L. & DAYAN, A. D. (1969) Rural Preponderance of Seminoma of the Testis. *Cancer, N.Y.*, **23**, 119.

SHARMA, K. C., GAETA, J. F., BROSS, I. D., MOORE, R. H. & MURPHY, G. P. (1972) Testicular Tumors. Histologic and Epidemiologic Assessment. *N.Y. State J. Med.*, **72**, 2412.